# Assessment of Attention Deficits in Adolescent Offspring Exposed to Maternal Type 1 Diabetes

**DOI:** 10.1371/journal.pone.0169308

**Published:** 2017-01-10

**Authors:** Birgitte Bytoft, Sine Knorr, Zuzana Vlachova, Rikke B. Jensen, Elisabeth R. Mathiesen, Henning Beck-Nielsen, Claus H. Gravholt, Dorte M. Jensen, Tine D. Clausen, Erik L. Mortensen, Peter Damm

**Affiliations:** 1 Center for Pregnant Women with Diabetes, Rigshospitalet, Copenhagen University Hospital, Copenhagen, Denmark; 2 Department of Obstetrics, Rigshospitalet, Copenhagen University Hospital, Copenhagen, Denmark; 3 Institute of Clinical Medicine, Faculty of Health and Medical Sciences, University of Copenhagen, Copenhagen, Denmark; 4 Department of Endocrinology and Internal Medicine, Aarhus University Hospital, Aarhus, Denmark; 5 Department of Molecular Medicine, Aarhus University Hospital, Aarhus, Denmark; 6 Department of Endocrinology, Odense University Hospital, Odense, Denmark; 7 Department of Clinical Research, Faculty of Health Sciences, University of Southern Denmark, Odense, Denmark; 8 Department of Growth and Reproduction, Rigshospitalet, Copenhagen University Hospital, Copenhagen, Denmark; 9 Department of Endocrinology, Rigshospitalet, Copenhagen University Hospital, Copenhagen, Denmark; 10 Department of Gynecology and Obstetrics, Odense University Hospital, Odense, Denmark; 11 Department of Gynecology and Obstetrics, Nordsjaellands Hospital, Hilleroed, Denmark; 12 Section of Environmental Health, Department of Public Health and Center for Healthy Aging, University of Copenhagen, Copenhagen, Denmark; University Children's Hospital Tuebingen, GERMANY

## Abstract

**Objective:**

The aim of this study was to examine the potential association between intrauterine exposure to maternal diabetes and attention deficits in the offspring.

**Research design and methods:**

Adolescent offspring of a prospectively followed cohort of women with type 1 diabetes (n = 269) and a control group from the background population (n = 293) participated in a follow-up assessment in 2012–2013. We used scores from Conners Continuous Performance Test II to assess attention and based on a principal component analysis we evaluated scores on five different attention factors: focused attention, vigilance, hyperactivity/impulsivity, sustained attention and response style.

**Results:**

A higher frequency of the exposed offspring had a parent/self-reported use of Attention Deficit Hyperactivity Disorder (ADHD) medication compared to the control group (2.2% vs. 0.0%, p = 0.01). Clinical significant differences between adolescents exposed to maternal diabetes and unexposed controls were not found in either single scores on Conners Continuous Performance Test or on any of the five attention factors identified.

**Conclusions:**

Exposure to maternal type 1 diabetes did not seem to increase the risk of attention deficits in the adolescent offspring. However, a higher self-reported use of ADHD medication in the exposed group could suggest a difference in attention not revealed by the applied test.

## Introduction

Attention-deficit hyperactivity disorder is a common neurodevelopmental disorder characterized by persistent hyperactivity, impulsivity and inattention with a worldwide prevalence of 3–4% [1–3]. The etiology of ADHD is complex and influenced by an interaction of multiple genetic and environmental factors [1, 4]. Possible pre- and perinatal risk factors are maternal obesity, maternal smoking and stress in pregnancy, prematurity, low birth weight and exposure to various toxins [5–7]. A few studies have suggested an association between exposure to maternal diabetes and later risk of attention deficits in the offspring [8–11].

We recently reported impaired long-term cognitive function in adolescent offspring of a well-characterized cohort of Danish women with type 1 diabetes [12]. Furthermore, we found increased frequencies of learning difficulties in primary school within the group of diabetes exposed offspring. Children with ADHD are known to have lower academic achievement and learning difficulties compared to unaffected children [1, 13], and abnormalities in various regions of the brain have been identified in these children [1, 14]. It is pivotal to identify individuals at risk of attention disorders at an early stage, since social problems, low educational and occupational achievements, criminal behavior and drug abuse are potential long-term adverse outcomes of ADHD [1, 15–18].

Animal studies as well as clinical studies have shown adverse effects of maternal diabetes on the development of the brain and behavior in the offspring [19–23], Thus it is possible that maternal diabetes is a causal risk factor of ADHD in the offspring.

We performed a follow-up examination of offspring of a well-characterized cohort of Danish women with type 1 diabetes to assess if exposure to maternal diabetes can affect the risk of attention deficits in adolescence.

## Research Design and Methods

EPICOM (EPIgenetic, genetic and environmental effects on COgnitive and Metabolic functions in offspring of mothers with type 1 diabetes) is a Danish prospective nation-wide follow-up study of the long-term effects of exposure to maternal diabetes.

During 1993–1999 all pregnant women with type 1 diabetes in Denmark were prospectively followed and data on maternal demography, diabetes status and pregnancy outcome were reported to a central register in the Danish Diabetes Association (n = 1215) [24]. The women delivered at one of eight centers responsible for antenatal care and deliveries of pregnant women with diabetes in Denmark at that time. All deliveries after 24 weeks were included.

### Participants

At the age of 13 to 19 years offspring of women in the register were invited to a follow-up examination (singletons and one child per mother) (n = 746). A control group matched according to gender, age and postal code (as a marker of socioeconomic status) was identified through the Danish Central Civil Registration System. At inclusion all control participants and/or their parents were asked about a prior history of gestational diabetes, and in addition all available obstetric records were scrutinized for information on gestational diabetes.

We excluded 16 diabetes exposed offspring due to: maternal diabetes diagnosis had been reclassified to MODY or type 2 diabetes (n = 12), no contact between mother and child (n = 2), drug abuse (n = 1), pregnancy at the time of recruitment (n = 1).

Reasons for exclusion of control offspring were: adopted child (n = 4), the obstetric record revealed a maternal diagnosis of gestational diabetes (n = 3), birth place outside of Denmark (n = 1).

**Fig 1:**
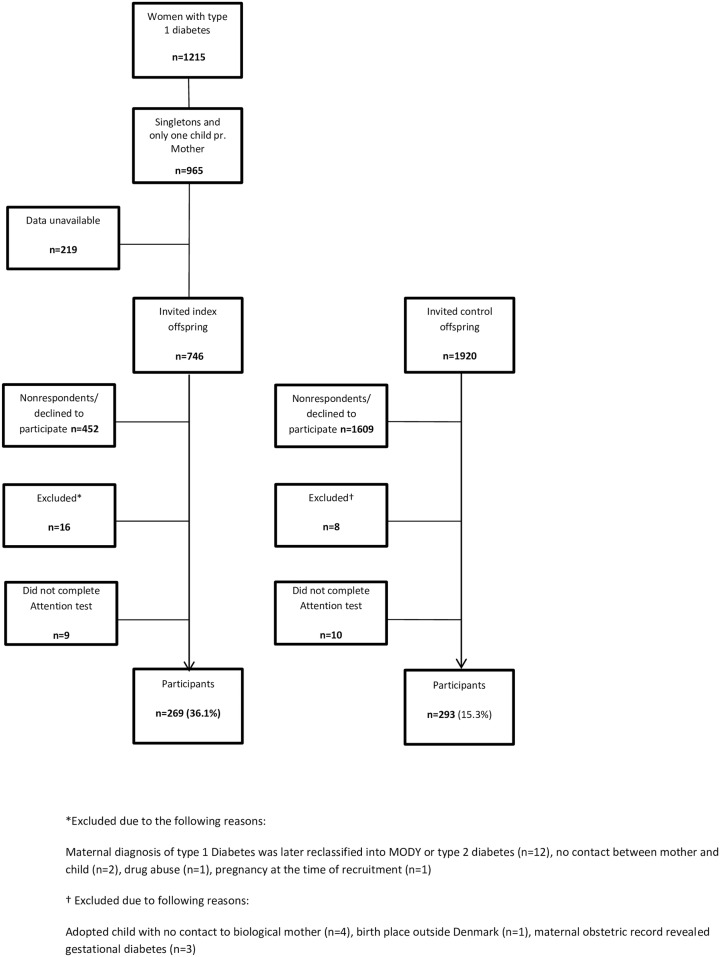
Flowchart of the study.

The EPICOM study examined 278 diabetes exposed offspring (exposed group, 37.1% of invited offspring) and 303 unexposed offspring (control group, 15.7% of invited offspring). Participants were all born in Denmark and mainly of white European ethnicity (98.8%). A total of 269 (36.1% of invited) exposed and 293 (15.3% of invited) control offspring completed the computer based attention test (Fig 1). All non-completions of the test (n = 9 in the diabetes exposed group, n = 10 in the control group) were due to technical difficulties related to computer problems. The inclusion process and the follow-up study have recently been described in detail [12, 25].

The protocol was in accordance with the Helsinki Declaration and approved by the regional ethical committee of Central Denmark (M-20110239). We obtained written informed consent from all participants or their parents if the participant was younger than 18 years.

#### Follow-up

Two diabetes exposed participants had known diabetes (type 1), and one was diagnosed with type 1 diabetes in our study sample. All participants, both diabetes exposed and controls, had an oral glucose tolerance test (OGTT) as a part of the examination program. Data from the OGTT has been published recently [25].

The participants were examined at one of three Danish University Hospitals (Copenhagen, Odense and Aarhus) in the period April 2012 to October 2013.

We used Conners Continuous Performance Test version II (Conners CPT-II, version 5 for Windows) [26] to evaluate attention. Continuous performance tests are a well-known and widely used test paradigm in the assessment of attention deficits [27]. Conners CPT-II is a high signal-to-noise test, where the participant has to respond to the majority of stimuli and repress the tendency to respond to the more infrequent non-targets. This implies a high demand on response inhibition, which is a typical deficit in ADHD [28].

In Conners CPT-II the participants had to press the spacebar for all letters, displayed on a computer screen, except when the letter X appeared. Administration time for the test is 14 minutes with six blocks of trials with various inter stimulus intervals (ISIs). ISIs are 1, 2 or 4 seconds and make it possible to test the ability to adjust to changing demands. All participants were given standardized instructions and completed a short practice session before the actual test, conducted by exposure blinded testers. The test was conducted after a light meal and in a quiet room without disturbances. Only the participant and the tester were present in the room during the test, while parents waited outside.

### Principal component analysis

Conners CPT-II provides a large number of individual test measures. To reduce the number of variables and to obtain a clearer picture of components of attention, we followed Egeland & Kovalik-Gran [29] and subjected the correlations among the different test performance indicators to a principal component analysis. Thus we analyzed T scores on the following variables: number of omissions, commissions, perseverations, hit reaction time, hit reaction time standard error (HR consistency), variability of standard errors or change in consistency style), hit reaction time by block (the slope of change in reaction time over six blocks), hit reaction time standard error by block (change in consistency over time), hit reaction time by differing inter-stimulus intervals and hit standard error by inter-stimulus interval (whether longer intervals are associated with more variance in performance) and response style. Like Egeland & Kovalic-Gran [29] we did not include detectability in the principal component analysis, but unlike these authors measures of change in omissions and commissions were not available for our analysis.

Only three factors had eigenvalues larger than 1, but varimax rotation with three, four and five retained components showed a pattern similar to that obtained by Egeland & Kovalic for only five components. Thus, the first five components explained 83% of the variance, and the rotated matrix of loading showed the following attention factors:

**Factor 1, Focused attention**: defined by the loadings of omissions, hit reaction time standard error, variability and perseverations. Factor 1 explained 24.0% of the variance.

**Factor 2, Vigilance**: defined by the loadings of hit reaction time by differing inter stimulus intervals and hit reaction time standard error by inter stimulus interval. Factor 2 explained 17.5% of the variance.

**Factor 3, Hyperactivity/impulsivity:** defined by the loading of commissions and hit reaction time. Factor 3 explained 16.1% of the variance.

**Factor 4, Sustained attention:** defined by the loadings of hit reaction time by block, hit reaction time standard error by block. Factor 4 explained 15.0% of the variance.

**Factor 5, Response style:** defined by the loading of response style. Factor 5 explained 10.3% of the variance.

The first four factors clearly corresponded to four of the Egeland & Kovalic factors with the same test scores having high loadings on the same factors except for the response style test score. Egeland & Kovalic called their fifth factor change in control and this fifth factor reflected the measures of change in omissions and commissions which were not included in our analysis. In their analysis Response Style loaded together with commissions and hit reaction time on the hyperactivity/impulsivity factors, but this was not the case in our analyses with rotation of 4 or 5 factors.

### Variables

#### Exposure

Exposure was maternal type 1 diabetes in fetal life.

#### Outcomes

Our primary outcomes were indications of attention deficits assessed by individual test measures in Conners CPT-II and by five attention factors derived from principal component analysis of variables from Conners CPT-II: focused attention, vigilance, hyperactivity/impulsivity, sustained attention and response style.

#### Maternal covariates

Parental educational level was estimated from a questionnaire completed by the offspring and their parents. It was calculated as the sum of maternal and paternal years in school and in higher education.

Maternal age at delivery, parity and information about complications in pregnancy were retrieved from the register in the Danish Diabetes Association (exposed group) or from obstetric medical records (control group). Pregnancy complications were defined as occurrence of hydramnios (clinical diagnosis) or preeclampsia (blood pressure>140/90 mmHg and proteinuria).

Information on breastfeeding, smoking in pregnancy and parental intelligence was not available. Sufficient data on delivery mode was not available in the control group and therefore not included in our analysis.

#### Offspring covariates

Information about gestational age, birth weight, congenital malformations and neonatal complications were provided from the Danish Diabetes Association register (exposed group) or retrospectively from obstetric medical records (control group).

We combined and defined neonatal complications as hypoglycemia (clinical signs disappearing after administration of glucose), jaundice (need of treatment with phototherapy), respiratory distress (need of assisted ventilation or continuous positive airway pressure>1 hour), Apgar 5min<7 and systemic infections (need of systemic antibiotics treatment).

Birth weight standard deviation scores (bwSDS) were calculated using intrauterine growth curves adjusted for gestational age and gender [30].

#### Potential mediators and confounders

We considered offspring gender, age at follow-up, parity, parental educational length and maternal age at delivery potential confounders.

Gestational age, bwSDS, pregnancy complications and neonatal complications were considered potential mediators between maternal diabetes and offspring attention deficits.

### Statistical analysis

Continuous data with normal distribution are presented as mean and standard deviation [31], while continuous data with skewed distribution are presented as median and interquartile range. Comparisons of groups were performed using student`s T-test, Mann-Whitney, Chi-squared and Fisher`s exact tests where appropriate.

We adjusted for confounders and mediators on each principal component factor in multivariate linear regression analyses with maternal diabetes as the independent variable and each of the principal component factors as outcome. The regression coefficient β with 95% confidence interval corresponds to the mean difference between the diabetes exposed and the control group.

Statistical analyses were performed with IBM statistics SPSS version 22, with a significance level of 0.05. Correction for multiple testing with the Bonferroni method was applied in analysis of our five principal components and in these tests the level of significance was 0.05/5 = 0.01.

## Results

Table 1 presents baseline and follow-up characteristics of the 269 (36.1% of the invited) exposed offspring and the 293 (15.3% of the invited) control offspring, who completed Conners CPT-II. Median age was 16.6 (range 15.3–18.0) and 17.0 (range 15.3–18.2) in the exposed and control group, respectively (P = 0.40). Differences between groups were found for gestational age, bwSDS, neonatal complications, neonatal hypoglycemia, congenital malformations, pregnancy complications and parity. At the time of follow-up the exposed group had a higher self-reported use of ADHD medication (2.2% vs. 0.0%, p = 0.01). These six participants had all been prescribed ADHD medication on the indication of ADHD, and the types of medication were: Methylphenidate (n = 3), Atomoxetine (n = 2) and Dexamfetamine (n = 1). Offspring gender, maternal age at delivery, maternal BMI and parental educational level did not differ between the groups.

**Table 1:** Characteristics of offspring exposed to maternal type 1 diabetes and a matched unexposed control group.

	Exposed	Unexposed	P
N	269 (36.1%)	293 (15.3%)	
Male gender	40.8% (111)	40.1% (118)	0.87
**Baseline**			
Gestational age (days)*	260 (251–266)	280 (274–287)	**<0.001**
Birth weight standard deviation score*	1.79 (0.41–3.27)	-0.05 (-0.64–0.61)	**<0.001**
Preterm delivery <34 weeks	9.9% (25)	0.5% (1)	**<0.001**
Preterm delivery <37 weeks	40.5% (104)	5.7% (6)	**<0.001**
Birth weight	3567 (796)	3556 (478)	0.857
Neonatal complications†	49.0% (125)	4.7% (9)	**<0.001**
Neonatal hypoglycemia	32.3% (84)	0.5% (1)	**<0.001**
Congenital malformations	3.8% (10)	0.0% (0)	**0.003**
Pregnancy complications‡	30.8% (80)	10.3% (19)	**<0.001**
Maternal age at delivery (years)	30.0 (4.1)	29.9 (4.1)	0.51
Nulliparity	59.8% (156)	42.1% (90)	**<0.001**
Maternal BMI (kg/m^2^)*	23.0 (21.3–25.2)	22.6 (20.6–24.8)	0.09
**Follow-up**			
Offspring age at follow-up*	16.6 (15.3–18.0)	17.0 (15.3–18.2)	0.40
Parental educational level#	27.6 (4.2)	28.2 (4.5)	0.16
Use of ADHD medication	2.2% (6)	0.0% (0)	**0.01**

Data are presented as mean (standard deviation) or proportions (n)

*Data are presented as median (25–75% percentiles), when not normally distributed

^†^ Hypoglycemia, jaundice, respiratory distress, Apgar5<7, systemic infection

^‡^ Hydramnios, preeclampsia

^#^ Sum of parental educational length in years

Mean maternal diabetes duration at conception in the exposed group was 12.5 (SD 8.2) years, and the frequencies of pregestational macroalbuminuri and proliferative retinopathy were 5.6% and 8.0%, respectively.

Participants and non-participants in the exposed group differed at baseline according to bwSDS (1.8 vs. 1.5 SDS, p = 0.02) and maternal peri-conceptional HbA1C (7.3% (56mmol/mol) vs. 7.5% (58 mmol/mol), p = 0.02), but were similar with respect to parity, maternal pre-pregnancy age, maternal pre-pregnancy BMI, duration of diabetes and HbA1C in second and third trimester.

### Attention scores

Differences between exposed and control offspring were not found in any of the individual test measures derived from Conners CPT II (Table 2). Similarly, the multivariate regression analysis of attention factors did not show significant differences between the two groups in either crude or adjusted analyses of attention factors 1 to 4. The exposed offspring had a marginally higher score on the fifth factor (Response style) in crude analysis (β = 0.169, p = 0.05) and in analyses adjusted for potential confounders (β = 0.238, p = 0.02) and mediators (β = 0.267, p = 0.05) (Table 3). Differences in the fifth factor were no longer significant after correction for multiple testing.

**Table 2:** T-scores in individual test measures in Conners Continuous Performance Test II. Offspring of women with type 1 diabetes (n = 269) compared to an unexposed control group (n = 293).

	Exposed	Unexposed	P
Omissions	51.1 (13.3)	49.6 (9.6)	0.12
Comissions	54.5 (10.4)	55.2 (11.2)	0.47
Hit RT	44.2 (8.7)	43.5 (9.2)	0.36
Hit RT standard error	45.0 (10.4)	44.6 (10.2)	0.64
Hit RT block change	48.5 (9.5)	48.6 (7.7)	0.88
Hit SE block change	51.9 (10.2)	52.8 (10.0)	0.30
Hit RT ISI change	47.0 (9.1)	46.9 (8.2)	0.90
Hit SE ISI change	47.5 (10.0)	48.1 (10.7)	0.46
Variability	45.9 (11.3)	45.9 (11.3)	0.99
Detectability	54.7 (8.8)	54.6 (8.5)	0.94
Response style	48.2 (7.8)	47.2 (6.4)	0.10
Perseverations	50.3 (10.1)	51.2 (13.1)	0.34

Data are presented as mean (standard deviation)

Hit RT = hit reaction time

Hit SE = hit standard error

ISI = inter stimulus interval

**Table 3:** Regression analyses of factor scores from Conners Continuous Performance Test-II. Offspring of women with type 1 diabetes (n = 269) compared to an unexposed control group (n = 293).

Factor	β	95% CI	P
**Focused attention**			
Crude	0.014	(-0.152 to 0.180)	0.87
Model 1	0.006	(-0.180 to 0.193)	0.95
Model 2	0.134	(-0.160 to 0.427)	0.37
**Vigilance**			
Crude	0.022	(-0.188 to 0.144)	0.80
Model 1	0.059	(-0.248 to 0.131)	0.54
Model 2	0.086	(-0.216 to 0.388)	0.58
**Hyperactivity/impulsivity**			
Crude	0.069	(-0.097 to 0.235)	0.42
Model 1	0.030	(-0.158 to 0.219)	0.76
Model 2	0.082	(-0.198 to 0.363)	0.56
**Sustained attention**			
Crude	0.052	(-0.218 to 0.114)	0.54
Model 1	0.009	(-0.202 to 0.184)	0.93
Model 2	0.090	(-0.219 to 0.400)	0.57
**Response style**			
Crude	0.169	(0.003 to 0.334)	0.05
Model 1	0.238	(0.046 to 0.430)	**0.02**
Model 2	0.267	(0.005 to 0.529)	0.05

Model 1: Potential confounders (offspring gender, age at follow-up, parity, parental educational length, maternal age at delivery)

Model 2: Potential mediators (gestational age, birth weight SDS, pregnancy complications and neonatal complications)

No interaction between gender and exposure was found. Gender differences were evaluated in a model controlling for exposure group and a higher male score was found for factor 3 (hyperactivity/impulsivity) (β = 0.33, p<0.001), but no significant differences in the remaining factors.

We did not observe any significant interaction between maternal diabetes and low parental education on the risk of attention deficits in the offspring.

We found low correlations between age at follow up and each of the five factors: correlation coefficients ranged from -0.13 to 0.16.

## Discussion

In this large prospectively followed cohort we found a higher self-reported use of ADHD medication, but no increase in attention deficits in adolescent offspring exposed to maternal type 1 diabetes in utero compared to a control group from the background population. We used Conners CPT-II to assess attention deficits and did not find any clinical significant differences on individual test measures or in crude or adjusted analysis of attention scores derived from principal component analysis. Attention scores were analyzed because combination of test scores may be more reliable and sensitive to attention deficits.

Focused attention (factor 1) is the ability to concentrate on a specific stimulus and exclude others. Vigilance (factor 2) is the ability to remain alert, when stimuli are less frequent. A high score on the factor hyperactivity/impulsivity (factor 3) are obtained with many commissions and short reaction time and would be expected in individuals with ADHD, while sustained attention (factor 4) describes the capacity to stay focused throughout the test and could be affected in individuals with depression [29]. A difference between our groups was found according to response style (factor 5), indicating a more cautious style in the exposed group. It should, however, be observed that the difference between the two groups was not significant when Bonferroni correction was applied to the results.

We performed analysis of the effects of gender and age, owing to a well-documented gender specific difference in attention deficits and remission of symptoms during adolescence [3]. Males had a higher score in one of the factors, hyperactivity/impulsivity, which is consistent with the evident overrepresentation of boys with attention deficits dominated by impulsivity and hyperactivity [32]. Correlations between offspring age and scores on the attention test were relatively weak in our cohort.

In a recently published article on the EPICOM cohort [12], offspring exposed to maternal diabetes had lower cognitive function and higher frequency of learning difficulties in the subjects Danish and mathematics in primary school compared to the control group. Since learning difficulties and reduced academic achievement among children with attention deficits are well-known [1, 33], a potential association between maternal diabetes and attention deficits in the offspring is plausible and has been suggested in previous studies [8–11]. However, this was not supported by the findings of the present study.

### Strengths and limitations

One of the major strength of this study is the use of a large, well-characterized and prospectively followed cohort of adolescent children of women with type 1 diabetes and a control group identified from the background population, both with detailed baseline information available.

In order to describe different aspects of attention we applied a high-signal-to-noise continuous performance test, a widely used test of attention deficits, in particular ADHD, and we identified attention factors in principal component analyses. Unfortunately, other sources of information on attention performance were not available. Impaired executive function and cognitive flexibility are known to be present in individuals with ADHD [28], and performance based tests of these functions as well as subjective ratings (parental and/or self-reported) of attention, impulsivity, executive function and cognitive flexibility would have enabled a more comprehensive assessment of attention deficits and cognition in our cohort. We were not able to specify the underlying diagnosis of the attention deficit (e.g. ADHD) with the applied test, since it would require a more comprehensive examination including diagnostic interviews and rating scales.

We did consider some of the potential problems with the use of Conners CPT II in our analyses, and chose not to include the overall confidence index. The identification of attention factors was done to focus on the different aspects of attention and to have a basis for deriving composite measures presumably more reliable than the individual test scores.

A limitation to our study was the risk of selection bias owing to the limited and different proportions of participation in our two groups. We have previously shown that both groups had intelligence scores above the population mean indicating selection from the better performing part of the population [12], which also might apply for the continuous performance test used to assess attention deficits. A systematic difference of participation between our groups would most likely be associated to positive selection of the control group, implying larger group differences. Thus, elimination of selection bias would result in differences supporting our null-hypothesis, which is equivalent to our conclusion.

Low socioeconomic status is known to have a strong effect on the risk of development of attention deficits in the offspring through different pathways [7]. The effects of maternal diabetes has been suggested to be more pronounced in low SES environments, which might explain the discrepancy between our findings and findings in similar studies due to a possibility of sample bias in our study [8–11]. Participation in a follow-up study with a comprehensive examination program requires personal resources and non-participation is in general associated with poorer health and lower socioeconomic status [34]. In our study the interaction between maternal diabetes and low parental educational level was not significant, and thus there was no evidence of stronger effects on attention in children from families with low parental educational level.

Finally, statistical power should be sufficient with 269 participants in the exposed group and 293 in the control group. Thus, the statistical power would be 84% for detecting a group difference of one quarter of a standard deviation.

### Other studies

In a nested case-control study, including individuals from the Norwegian Birth and Prescription Databases, only 3.0% (n = 88) of diabetes exposed offspring had been prescribed ADHD medication in the period 2004–2012 [8]. However, offspring exposed to maternal type 1 diabetes had higher odds of ADHD (OR = 1.7, 95% CI = 1.3–2.1) in models adjusted for parental diagnosis of ADHD and perinatal and socioeconomic confounders. We found a difference between our groups in the use of ADHD medication: 2.2% of the diabetes exposed offspring and none in the control group had self-reported use of ADHD medication at the time of follow-up. This could indicate a difference in attention deficits not revealed by administration of Conners CPT-II. Medication might reduce symptoms in the affected individuals and modify test scores towards the normal range. Sample bias with selection of the better performing part of the population in both groups is supported by these numbers, as the population prevalence of attention deficits has been estimated to 3–4% [1]. The controls represent the background population and we would expect some individuals with attention deficits in this group. Both groups could include individuals with attention deficits without current use of ADHD medication. We did not have permission to corroborate the self-reported information with information from the Danish National Prescription Register or from the National Patient Register on admissions to psychiatric departments.

Increased risk of ADHD symptoms in offspring exposed to maternal gestational diabetes (GDM) has been described previously [9, 10], in particular when GDM was present together with low socioeconomic status. One study combined a battery of neuropsychological tests with parental interviews on 212 preschool children (GDM offspring, n = 21) in New York and found a small increased risk in those exposed to maternal GDM. When GDM was present with low socioeconomic status the risk of ADHD at age six was synergistically increased suggesting an interaction of these risk factors [10]. Similar findings are reported in a German nationwide survey of children and adolescents [9].

In line with this, a register-based study on the EPICOM cohort found that offspring born to mothers with poor glycemic control in pregnancy attained lower grades in primary school compared to matched controls, while those born to mother with good glycemic control obtained better grades than their matched controls in an analysis adjusted for parental educational level [35]. Achievement of good glycemic control and management of a complex chronic disease are related to a high level of personal and social resources, which most likely will be reflected in the parenting role and upbringing of the child. However, in this study of attention deficits, interaction between parental educational level and exposure to maternal diabetes did not affect measures of attention in the offspring.

It is possible that not only intrauterine exposure to diabetes but also a shared genetic susceptibility to both type 1 diabetes and ADHD could influence attention in the offspring. A German register study found an increased frequency of ADHD in children with type 1 diabetes compared to those without, while a Norwegian study found no association between type 1 diabetes and ADHD [36, 37]. Offspring of a mother with type 1 diabetes have a diabetes lifetime risk of 3% [38], and we included three diabetes exposed offspring with type 1 diabetes in our cohort. All offspring without a prior diabetes diagnosis completed an oral glucose tolerance test at the day of cognitive testing, ensuring that all individuals with diabetes should be identified. With only three cases of type 1 diabetes genetic association should only be a minor contributor to our results.

## Conclusions

In conclusion, we found a higher self-reported use of ADHD medication among diabetes exposed offspring compared to the control group. However, no increased risk of attention deficits in adolescence was demonstrated in this large, prospective and well-characterized cohort exposed to maternal Type 1 diabetes during fetal life.
